# Gestational diabetes mellitus in the COVID‐19 pandemic: A retrospective study from Hangzhou, China

**DOI:** 10.1111/1753-0407.13324

**Published:** 2022-10-01

**Authors:** Binbin Yin, Kaiqi Wu, Lingwei Hu, Wanlu Zheng, Yidan Zheng, Xiuzhi Duan, Bo Zhu

**Affiliations:** ^1^ Department of Laboratory Medicine The Women's Hospital of Zhejiang University School of Medicine Hangzhou China; ^2^ Department of Genetics and Metabolism The Children's Hospital of Zhejiang University School of Medicine Hangzhou China; ^3^ Department of Laboratory Medicine The Second Affiliated Hospital of Zhejiang University School of Medicine Hangzhou China

**Keywords:** COVID‐19, gestational diabetes mellitus, pregnancy outcomes, prevalence, COVID‐19, 妊娠糖尿病, 患病率, 妊娠结局

## Abstract

**Aims:**

Our study aimed to investigate changes in the prevalence of gestational diabetes mellitus (GDM) in the COVID‐19 pandemic and postpandemic era and the risk of adverse pregnancy outcomes in pregnant women diagnosed with GDM during the blockade period.

**Methods:**

First, we investigated changes in the prevalence of GDM and the population undergoing oral glucose tolerance tests (OGTT) after the COVID‐19 pandemic. We then collected clinical information from pregnant women diagnosed with GDM to explore the risk of adverse pregnancy outcomes in pregnant women with GDM during the COVID‐19 pandemic.

**Results:**

After the COVID‐19 pandemic, the proportion of pregnant women in the total number of outpatient OGTT tests decreased yearly. The ratio was 81.30%, 79.71%, and 75.48% from 2019 to 2021, respectively, with the highest proportion of pregnant women in February 2020 (92.03%). The prevalence of GDM was higher in March 2020 compared to the same period in 2019. However, from 2019 to 2021, the prevalence decreased year by year with 21.46%, 19.81%, and 18.48%, respectively. The risk of adverse pregnancy outcomes for pregnant women diagnosed with GDM during the most severe period of the COVID‐19 pandemic did not differ from before the COVID‐19 pandemic.

**Conclusions:**

After the COVID‐19 pandemic, the prevalence of GDM increased during the most severe period of the epidemic, but the overall prevalence of GDM decreased year by year. In addition, the pandemic did not change the risk of adverse pregnancy outcomes in pregnant women with GDM.

## Graphical abstract

**Highlights**

- The COVID‐19 pandemic affected nonpregnant women who had oral glucose tolerance tests in outpatient clinics more than pregnant women.
- After the COVID‐19 pandemic, the prevalence of gestational diabetes mellitus (GDM) increased at the height of the epidemic, but the overall prevalence of GDM decreased yearly from 2019 to 2021.
- The risk of adverse pregnancy outcomes in pregnant women with GDM after the COVID‐19 pandemic did not differ from before the pandemic.

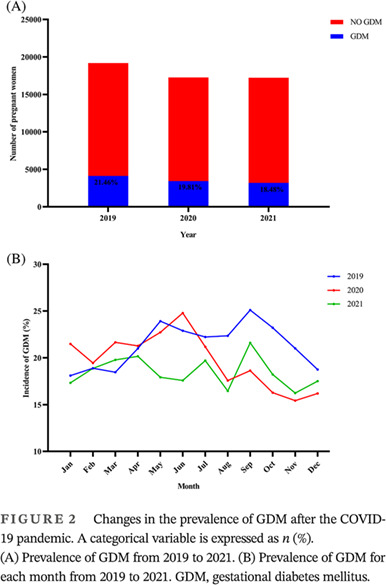

## 摘要

**目的:**

我们的研究旨在调查 COVID‐19 大流行和大流行后时代妊娠糖尿病(GDM)患病率的变化, 以及在封锁期间诊断为GDM的孕妇发生不良妊娠结局的风险。

**方法:**

首先, 我们调查了 COVID‐19 大流行后 GDM 患病率和接受口服葡萄糖耐量试验 (OGTT) 的人群的变化。然后, 我们收集了被诊断患有 GDM 的孕妇的临床信息, 以探讨在 COVID‐19 大流行期间患有 GDM 的孕妇出现不良妊娠结局的风险。

**结果:**

在 COVID‐19 大流行之后, 孕妇在门诊 OGTT 检测总数中的比例逐年下降。 2019年至2021年该比例分别为81.30%、79.71%和75.48%, 2020年2月孕妇比例最高(92.03%)。 2020年3月GDM患病率高于2019年同期, 但2019‐2021年患病率逐年下降, 分别为21.46%、19.81%和18.48%。在 COVID‐19 大流行最严重时期诊断为 GDM 的孕妇的不良妊娠结局风险与 COVID19 大流行之前没有差异。

**结论:**

COVID‐19大流行后, GDM患病率在疫情最严重时期呈上升趋势, 但GDM总体患病率逐年下降。此外, 大流行并未改变 GDM 孕妇不良妊娠结局的风险。

## 1 INTRODUCTION

Gestational diabetes mellitus (GDM) is the first occurrence of glucose intolerance during pregnancy and is one of the most common pregnancy complications.^1^ The overall prevalence of GDM in China was 14.8%, which means that many pregnant women in China suffer from GDM.^2^ This prompts us to pay more attention to the prevention and control of GDM and identify potentially modifiable risk factors associated with the risk of developing GDM. The COVID‐19 pandemic has changed how we work, exercise, live, and learn while also causing damage to human health since its outbreak at the end of 2019.^3^, ^4^ The COVID‐19 pandemic has made people emotionally sensitive, especially pregnant women, who are more emotionally sensitive than the general population and are at higher risk of depression.^5^ A history of depression is significantly associated with an increased risk of GDM, implying that mood may be a modifiable risk factor for GDM.^6^, ^7^ In addition, the isolation measures taken in response to the COVID‐19 pandemic have affected people's lifestyles. For example, during the COVID‐19 pandemic, people stayed home and visited the doctor less often to maintain social distance. A UK study found that the COVID‐19 pandemic made 79% of women more sedentary, whereas the percentage of those who met activity guidelines fell to 23% from 47% before the pandemic.^8^ The COVID‐19 pandemic has affected access to fresh foods.^9^ However, a healthy plant‐based diet and lifestyle may help reduce the risk of developing severe COVID‐19 and death, as well as the risk of GDM.^10^, ^11^

The impact of the COVID‐19 pandemic on pregnant women, such as psychological factors (depression), lack of exercise, unhealthy diet, and a sedentary lifestyle, are all risk factors for GDM.^12^, ^13^ The confinement measures taken in response to the COVID‐19 pandemic had a negative impact on pregnant women, for example, with a significantly higher incidence of GDM.^14^, ^15^ Similarly, a recent study from Guangdong, China, found that the COVID‐19 pandemic increased the prevalence of GDM (15.2% vs. 12.4%).^16^ Moreover, the COVID‐19 pandemic has worsened postprandial glycemic control in patients with GDM, leading to an increase in the proportion of pregnant women requiring insulin.^17^

China controlled the spread of the epidemic as early as April 2020 and entered a “dynamic COVID‐zero” period.^18^ Current studies have focused on the short‐term changes of the COVID‐19 pandemic lockdown period on the prevalence of GDM.^14^, ^16^, ^17^, ^19^ However, no study has examined differences in the prevalence of GDM after control of the COVID‐19 pandemic and the risk of pregnancy outcomes in pregnant women diagnosed with GDM during the pandemic blockade. Therefore, this study sought to investigate the changes of the COVID‐19 pandemic and postpandemic era on the prevalence of GDM and the risk of adverse pregnancy outcomes of pregnant women diagnosed with GDM during the lockdown period of the COVID‐19 pandemic.

## 2 METHODS

### 2.1 Participants and study design

The Zhejiang Provincial Health Planning Commission announced the first local case of COVID‐19 in Zhejiang Province on 23 January 2020, and initiated a Level 1 response to a major public health emergency. The response became Level 3 until 23 March 2020. During a Level 1 response, some public places are temporarily closed, such as shopping centers and bars; social events and gatherings for residents are restricted.^16^ During the Level 3 response, public places take routine preventive measures such as wearing masks, taking body temperature, and maintaining social distancing. As a result, life gradually returns to what it was before the COVID‐19 pandemic. As of 13 April 2020, no local cases were reported in Zhejiang Province, and no local cases were reported in Hangzhou on 20 February 2020.^20^

We retrospectively analyzed pregnant women who underwent oral glucose tolerance tests (OGTT) from 2019 to 2021 at the Women's Hospital of Zhejiang University School of Medicine. Our hospital is the largest obstetrics and gynecology specialty hospital in Zhejiang Province, with nearly 20 000 deliveries per year. The Ethics Committee of the Women's Hospital of Zhejiang University School of Medicine approved our study (IRB‐20220103‐R). From 2019 to 2021, the same methods and criteria were used to diagnose GDM. For example, an OGTT was performed between 24 and 28 weeks of pregnancy. According to the International Association of Diabetes and Pregnancy Study Groups, GDM is diagnosed when any 75 g OGTT glucose value is reached or exceeded (fasting glucose, 1 and 2 h glucose ≥92, 180, and 153 mg/dl, respectively).^1^ To ensure the precision and accuracy of the test results, our laboratory has to perform internal quality control every day, and only after the internal quality control is passed can the samples be tested. The Westgard multirule quality control method for internal quality control has a long‐term cumulative coefficient of variation of less than 1.38%. Furthermore, in addition to the internal quality control, our laboratory participates in the external quality assurance scheme in Zhejiang Center for Clinical Laboratories and National Center for Clinical Laboratories and has achieved excellent results.

We then collected clinical information and pregnancy outcomes from pregnant women diagnosed with GDM in the first year of the COVID‐19 pandemic (January to February 2020), the same period before the COVID‐19 pandemic (January to February 2019), and the second year of the COVID‐19 pandemic (January to February 2021). We extracted participant information from the hospital's electronic medical system. The details are as follows: maternal age, prepregnancy weight, infant weight, height, gestational age, laboratory indicators, and neonatal outcomes. A total of 1436 pregnant women were diagnosed with GDM. Of these women, 156 had no pregnancy outcome, 70 had multiple pregnancies, 4 had miscarriages, and 2 had stillbirths. Finally, there were 1204 pregnant women included in the study.

### 2.2 Statistical analysis

We analyzed the data using IBM SPSS (version 23.0). Data are expressed as the mean ± SD for continuous variables, and number and percentage (%) are used for categorical variables. The Student's *t* test analyzed differences in continuous variables, and the Pearson χ^2^ test analyzed categorical variables. The effect of the COVID‐19 pandemic on adverse pregnancy outcomes in pregnant women with GDM was calculated by odds ratios or adjusted odds ratios with 95% confidence intervals. Our analysis adjusted confounding variables, such as age, preconception body mass index (BMI), birth weight, and fasting blood glucose. Therefore, a *p* value of <.05 was considered to be statistically significant.

## 3 RESULTS

### 3.1 Impact of the COVID‐19 pandemic on patients undergoing OGTT in the outpatient clinic

**FIGURE 1:**
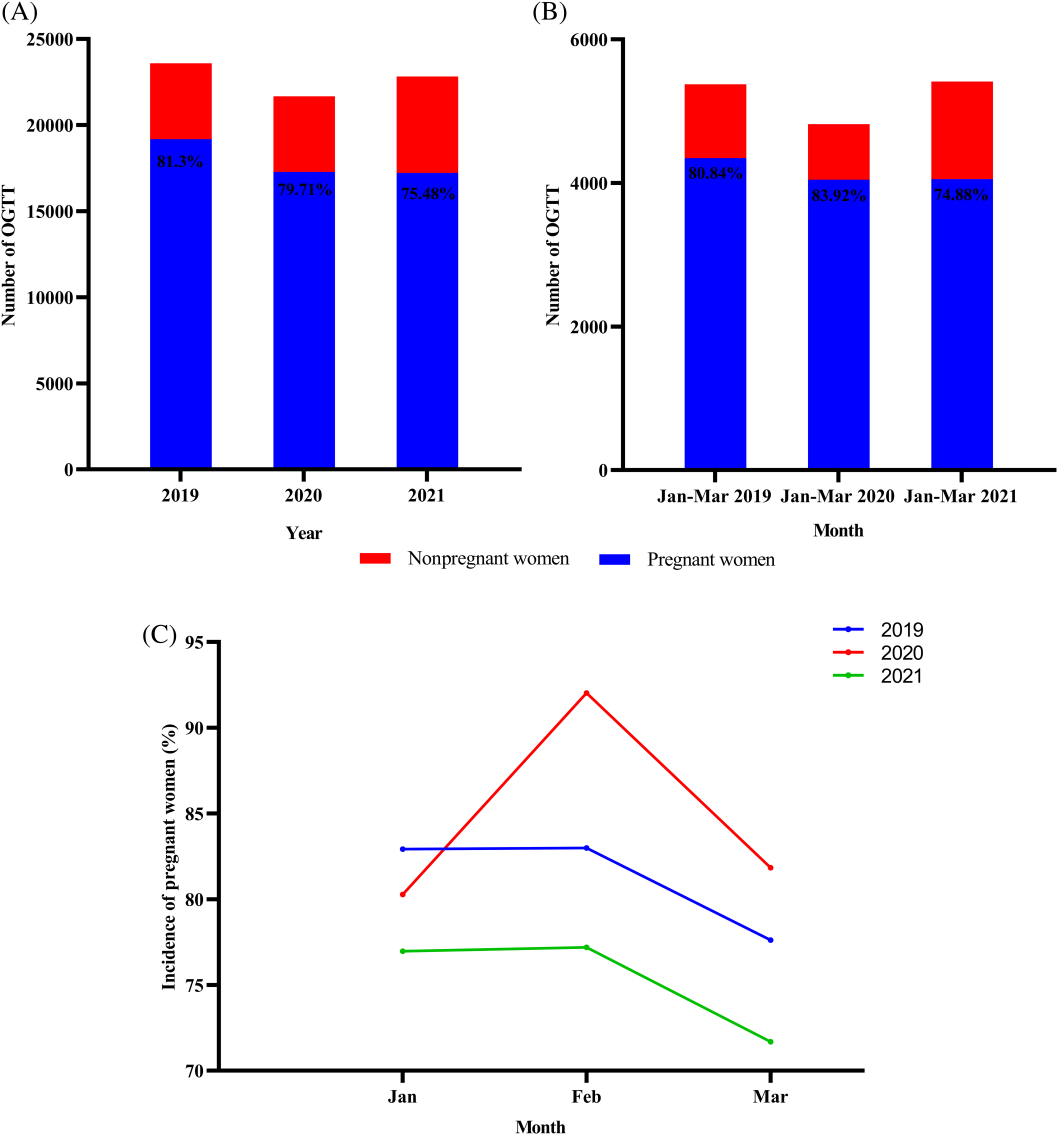
Impact of the COVID‐19 pandemic on patients undergoing OGTT in the outpatient clinic. A categorical variable is expressed as *n* (%). (A) The number of OGTT trials performed in outpatient clinics and the proportion of pregnant women from 2019 to 2021. (B) The number of OGTTs performed in outpatient clinics and the proportion of pregnant women from January to March (2019 to 2021). (C) The proportion of pregnant women in the number of outpatient OGTT trials performed from January to March (2019 to 2021). OGTT, oral glucose tolerance test.

The total number of OGTT performed each year after the COVID‐19 outbreak was less than in 2019 (Figure 1A). Although the number of tests in 2021 increased compared to 2020, the number of pregnant women receiving OGTT in the outpatient clinic did not change significantly and was significantly less than in 2019. As shown in Figure 1A, the proportion of pregnant women among the total number of people receiving OGTT in outpatient clinics continues to decrease from 2019 to 2021, at 81.30%, 79.71%, and 75.48%, respectively. However, the percentage of pregnant women who received OGTT as outpatients was 83.92% from January to March 2020 (the COVID‐19 pandemic period), significantly higher than in the same period in 2019 and 2021 (Figure 1B). February 2020, the worst month of the COVID‐19 pandemic, saw the highest percentage of pregnant women, 92.03%, of the total number of people receiving OGTT in the outpatient clinic (Figure 1C).

### 3.2 Changes in the prevalence of GDM after the COVID‐19 pandemic

**FIGURE 2:**
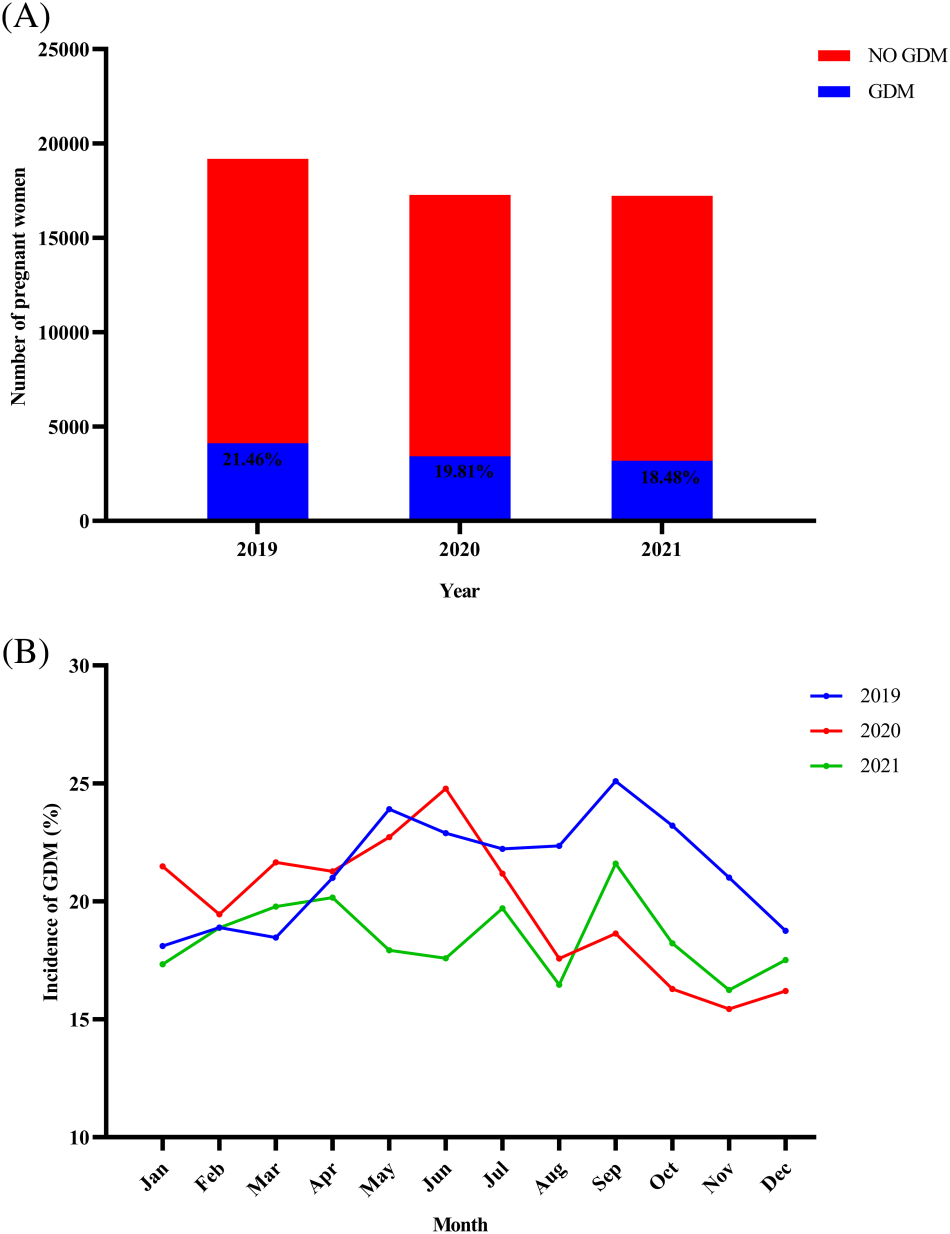
Changes in the prevalence of GDM after the COVID‐19 pandemic. A categorical variable is expressed as *n* (%). (A) Prevalence of GDM from 2019 to 2021. (B) Prevalence of GDM for each month from 2019 to 2021. GDM, gestational diabetes mellitus.

As shown in Figure 2A, the prevalence of GDM decreases each year after the COVID‐19 pandemic, with 21.46%, 19.81%, and 18.48% from 2019 to 2021, respectively. Figure 2B and Table 1 show the prevalence of GDM for each month from 2019 to 2021. Compared to the GDM prevalence rate in the same period in 2019 before the COVID‐19 pandemic, it was elevated in January and March and decreased from August to November 2020, with no difference at other times. In addition, GDM prevalence was lower in 6 months in 2021 (May, June, August, September, October, and November) than in 2019, with no differences in GDM prevalence in the other 6 months. Compared to the prevalence of GDM in the same period in 2020, the prevalence in 2021 increased in September; decreased in January, May, and June; and did not differ in the other times.

**TABLE 1:** Monthly prevalence of GDM, from 2019 to 2021

Time	2019		2020		2021		*p* 2019 vs 2020	*p* 2019 vs 2021	*p* 2020 vs 2021
	GDM/*N*	GDM (%)	GDM/*N*	GDM (%)	GDM/*N*	GDM (%)			
January	285/1574	18.11	272/1266	21.48	231/1333	17.33	.024	.585	.007
February	212/1122	18.89	220/1131	19.45	216/1144	18.88	.737	.993	.730
March	305/1651	18.47	357/1649	21.65	312/1577	19.78	.023	.344	.192
April	340/1620	20.99	322/1514	21.27	285/1414	20.16	.848	.572	.458
May	377/1577	23.91	329/1448	22.72	275/1535	17.92	.441	<.001	.001
June	402/1756	22.89	380/1534	24.77	266/1512	17.59	.207	<.001	<.001
July	426/1917	22.22	355/1676	21.18	315/1599	19.70	.450	.068	.293
August	409/1830	22.35	244/1388	17.58	267/1622	16.46	.001	<.001	.415
September	425/1694	25.09	267/1433	18.63	327/1514	21.60	<.001	.02	.045
October	356/1534	23.21	233/1431	16.28	250/1372	18.22	<.001	.001	.174
November	313/1490	21.01	218/1412	15.44	215/1324	16.24	<.001	.001	.567
December	265/1413	18.75	226/1395	16.20	224/1279	17.51	.075	.404	.365
Total	4115/19 178	21.46	3423/17 277	19.81	3183/17 225	18.48	<.001	<.001	.002

*Note*: Categorical variables were tested using Pearson's Chi‐square test or Fisher's exact test.

Abbreviations: GDM, gestational diabetes mellitus.

### 3.3 Clinical characteristics of pregnant women with GDM

A total of 1436 pregnant women who underwent OGTT in January to February of each year from 2019 to 2021 were diagnosed with GDM, of whom 1204 were included in the study. Table 2 demonstrates the pregnant women's clinical characteristics in the included research. There were no differences in the clinical features of pregnant women included in the study in 2020 compared to 2019, before the COVID‐19 pandemic. However, the maternal age, BMI, birth weight, and fasting glucose of pregnant women with GDM included in the study in 2021 were lower than in 2019.

**TABLE 2:** Clinical characteristics of pregnant women with GDM included in the study

Maternal characteristics	January to February 2019 (*N* = 418)	January to February 2020 (*N* = 401)	January to February 2021 (*N* = 385)	*p* (2019 vs 2020)	*p* (2019 vs 2021)
Maternal age, years	33.25 ± 4.45	33.00 ± 4.24	32.17 ± 4.19	.399	.001
BMI, kg/m^2^	22.23 ± 3.19	21.93 ± 3.37	21.53 ± 3.14	.185	.002
Gestational weight gain, kg	12.84 ± 4.06	12.48 ± 4.05	12.57 ± 3.82	.210	.331
Gestational age, week	38.43 ± 1.53	38.53 ± 1.38	38.28 ± 1.63	.314	.196
Birth weight, g	3327.99 ± 473.05	3307.84 ± 469.56	3201.07 ± 501.09	.541	.001
Parity, yes	150 (35.88%)	154 (38.40%)	139 (36.10%)	.456	.949
FPG, mmol/L	4.87 ± 0.53	4.81 ± 0.60	4.78 ± 0.51	.145	.011
1‐h GLU, mmol/L	9.98 ± 1.68	10.10 ± 1.60	10.02 ± 1.46	.304	.727
2‐h GLU, mmol/L	8.67 ± 1.53	8.70 ± 1.63	8.79 ± 1.41	.836	.255

*Note*: Continuous variables were tested using a *t* test with two independent samples; Categorical variables were tested using Pearson's chi‐square test or Fisher's exact test.

Abbreviations: BMI, body mass index; FPG, fasting plasma glucose; GDM, gestational diabetes mellitus; GLU, glucose.

### 3.4 Association of COVID‐19 pandemic on pregnancy outcomes in pregnant women with GDM

The impact of COVID‐19 on pregnancy outcomes in pregnant women with GDM is shown in Table 3. There was no difference in the risk of adverse pregnancy outcomes for pregnant women with GDM after the COVID‐19 pandemic compared to 2019.

**TABLE 3:** Effect of COVID‐19 on pregnancy outcome in pregnant women with GDM

	January to February 2019		January to February 2020			January to February 2021		
	*N* (%)	OR	*N* (%)	AOR (95% CI)	*p*	*N* (%)	AOR (95% CI)	*p*
Macrosomia^a^	30 (7.18)	1	25 (6.23)	0.87 (0.50–1.52)	.625	14 (3.64)	0.54 (0.28–1.03)	.063
LGA	53 (12.68)	1	44 (10.97)	0.98 (0.52–1.85)	.951	40 (10.39)	1.71 (0.89–3.30)	.102
SGA	22 (5.26)	1	22 (5.49)	0.99 (0.48–2.06)	.981	33 (8.57)	1.09 (0.57–2.09)	.795
Preterm birth	33 (7.89)	1	27 (6.73)	0.76 (0.39–1.47)	.416	32 (8.31)	0.76 (0.39–1.48)	.419
Cesarean delivery	201 (48.09)	1	172 (42.89)	0.85 (0.64–1.14)	.283	178 (46.23)	1.09 (0.81–1.47)	.577
Preeclampsia	44 (10.53)	1	46 (11.47)	1.16 (0.74–1.87)	.498	37 (9.61)	0.97 (0.60–1.59)	.916

*Note*: Adjusted for maternal age, preconception BMI, birth weight, and FBG.

Abbreviations: AOR, adjusted odds ratio; BMI, body mass index; CI, confidence interval; FBG, fasting blood glucose; GDM, gestational diabetes mellitus; LGA, large for gestational age; OR, odds ratio; SGA, small for gestational age.

^a^ Adjusted for maternal age, preconception BMI, and FBG.

## 4 DISCUSSION

Our study aimed to assess changes in the prevalence of GDM and the risk of adverse pregnancy outcomes in pregnant women with GDM in the COVID‐19 pandemic and postpandemic era. We found the following: (a) COVID‐19 pandemic affects nonpregnant women in outpatient visits more than pregnant women; (b) After the COVID‐19 pandemic, the prevalence of GDM increased during the most severe period of the epidemic, But the overall prevalence of GDM showed a decreasing trend from 2019 to 2021, with 21.46%, 19.81%, and 18.48%, respectively; and (c) The risk of adverse pregnancy outcomes in pregnant women with GDM after the COVID‐19 pandemic did not differ from before the pandemic.

The COVID‐19 pandemic puts a significant burden on health agencies by requiring more effort and time from medical staff to prevent the spread of the epidemic. Therefore, medical resources available for maternal care may have declined from levels before the COVID‐19 pandemic, resulting in some routine prenatal care being delayed or eliminated. Maternal health service use decreased significantly in 36 of 37 low‐ and middle‐income countries during the COVID‐19 pandemic in 2020.^21^ A study in China also showed a significant reduction in outpatient visits, emergency department visits, and hospitalizations following the COVID‐19 pandemic compared to the same period in 2019.^22^ In addition to the decrease in medical resources, it is more because pregnant women fear infection and reduce the frequency of antenatal visits. For example, our study found that after the COVID‐19 outbreak, the proportion of pregnant women among those receiving OGTT in the outpatient clinic decreased year by year to 81.3%, 79.71%, and 75.48%, respectively. Although the number of tests in 2021 is significantly higher than in 2020 and closer to 2019, the number of pregnant women in it is little changed and considerably less than in 2019. These results imply that pregnancies may be declining after the COVID‐19 pandemic. Some studies have found that releasing China's “universal two‐child policy” did not result in the expected “baby boom.”^23^, ^24^ Moreover, facing a COVID‐19 pandemic outbreak may lead to a further decline in fertility levels. In January to March 2020, although the number of pregnant women undergoing OGTT in the outpatient clinic did not change significantly, the percentage reached 83.92%, substantially higher than in the same period in 2019 and 2021. In addition, there was no significant change in the number of pregnant women receiving OGTT in an outpatient setting at the start of the COVID‐19 pandemic in February 2020. However, this month had the highest percentage of pregnant women (92.03%), much higher than the same period in 2019 and 2021. Our data show a significant decrease in nonpregnant patients undergoing OGTT after the COVID‐19 epidemic but no change in the number of pregnant women, suggesting that the COVID‐19 epidemic had a more significant impact on nonpregnancy. This is easily explained by the fact that women who are not pregnant undergo OGTT mainly because of infertility, polycystic ovarian syndrome, or obesity, which are not urgently needed to be addressed and are therefore pushed back or through telemedicine consultations. For example, during the COVID‐19 pandemic, gynecologic telemedicine services expanded rapidly, accounting for 94% of general consultations.^25^

The mechanism of new‐onset diabetes in COVID‐19 patients is unclear. It may have the following causes: stress hyperglycemia, steroid hormone‐induced hyperglycemia, previously undiagnosed diabetes, and impaired glucose handling and insulin secretion.^26^ A recent study found that the first glucose after hospitalization in elderly COVID‐19 patients showed that 20.8% were newly diagnosed with diabetes, and 28.4% were diagnosed with abnormal glucose.^27^ Moreover, the prevalence of GDM was significantly higher during the COVID‐19 pandemic in 2020 than in 2019 (9% vs. 13.5%).^14^ Studies from China also suggest that the COVID‐19 pandemic may increase the risk of GDM.^16^, ^19^ However, our findings indicate that the short‐ and long‐term effects of the COVID‐19 pandemic on the prevalence of GDM may not be consistent. During the most severe period of the COVID‐19 pandemic, the prevalence of GDM was higher only in January and March 2020 than in the same period of 2019 before the COVID‐19 outbreak. However, there was no difference in the prevalence of GDM in February, probably because the COVID‐19 pandemic had just started and did not affect this group of pregnant women. In addition, we do not believe that the elevated prevalence of GDM in January 2020 is associated with the COVID‐19 pandemic. This is because the measures taken in response to the COVID‐19 pandemic were released in late January. Our results found a significantly higher prevalence of GDM in March (21.65% vs. 18.47%), which may be related to the COVID‐19 pandemic. The impact of the COVID‐19 pandemic on pregnant women, such as psychological factors (depression during pregnancy), lack of exercise, and sedentary lifestyle, are all risk factors for GDM.^12^

Women are more susceptible to environmental influences during pregnancy, and the COVID‐19 pandemic has increased their depression and anxiety.^28^ Depressive symptoms in early pregnancy are associated with an increased risk of GDM.^29^ Depression can activate the hypothalamic–pituitary–adrenal axis, leading to enhanced and sustained cortisol, which has an anti‐insulin effect, ultimately leading to GDM.^30^, ^31^ An Italian study found a significant increase in the prevalence of GDM during the COVID‐19 pandemic, which the authors attributed to the stress caused by the COVID‐19 pandemic leading to chronic inflammation, which increases the risk of GDM.^14^ In addition, exercise during pregnancy (from week 10 to week 14) can reduce the prevalence of GDM.^32^ An online survey during the COVID‐19 pandemic found that pregnant women who met physical activity guidelines dropped from 47% before the COVID‐19 pandemic to 23%, primarily because of fear of leaving home due to the COVID‐19 pandemic.^8^ These studies laterally support our results that the prevalence of GDM was higher during the worst period of the COVID‐19 pandemic (March 2020) than during the same period in 2019 and 2021. A study from China enrolling pregnant women who delivered in June to July 2020 (control group) and October to December 2020 (exposed group) found an increased risk of GDM during the COVID‐19 pandemic, which is the same as the results of our partial study.^19^ In contrast, our study recruited pregnant women after the COVID‐19 pandemic and during the same period in the past for comparison, which is more scientific and meaningful. We must also consider the effect of temperature and season on the prevalence of GDM.^33^, ^34^, ^35^

We found an increase in the prevalence of GDM in March 2020, but with a different outcome in the long term. From 2019 to 2021, the prevalence of GDM decreased year by year with 21.46%, 19.81%, and 18.48%, respectively. This may be because China successfully controlled the spread of the epidemic as early as April 2020, entering a “dynamic COVID‐zero” period with no lasting impact on pregnant women. After the COVID‐19 pandemic outbreak, to provide scientific and standardized interventions for pandemic‐related psychological problems, the Chinese government took timely measures to promote and educate the public on mental health and make full use of online communication to provide 24/7 psychological services. A study investigating psychological changes in the first wave of the COVID‐19 pandemic (mid‐January 2020 to end‐March 2020) found that anxiety levels tended to rise and then fall, suggesting that psychological problems caused by the COVID‐19 pandemic gradually receded over time and with the control of the pandemic.^28^ There may be several reasons for this. First, pregnant women received more companionship and emotional support from their families during the pandemic. Second, they feel safe with actions taken by the government, such as free vaccinations and viral nucleic acid testing. Last, giving pregnant women easily accessible, safe, and satisfactory medical care can reduce their anxiety.^36^ For example, hospitals offer convenient, round‐the‐clock emergency services for pregnant women, setting up separate access lanes, visitor restrictions, and environment disinfection. The online survey of pregnant women found that 47.3% of participants believed they were unlikely to be infected with COVID‐19.^37^ The government and hospitals have taken sufficient measures to control the spread of the COVID‐19 outbreak and its effects, so the impact on pregnant women is limited.

As we know, regular physical activity enhances insulin sensitivity and beta‐cell function and promotes increased glucose uptake by active skeletal muscle via pathways other than insulin.^38^, ^39^ After the COVID‐19 pandemic, the uncertainty of the pandemic has led to a decrease in outdoor exercise for pregnant women. But home exercise, such as housework, cooking food, playing with children, and online fitness classes, may increase. Before the COVID‐19 pandemic, fitness classes were not an option for pregnant women to exercise because of the many obstacles that pregnant women had to overcome, such as work, family responsibilities, and completing at a set time and place. However, the popular online fitness classes during the postpandemic era allowed pregnant women to choose a physical activity they had not previously participated in, ensuring they could exercise adequately at home. In addition, the diet during the COVID‐19 pandemic and postpandemic era showed some “favorable” changes, such as increased home cooking and more opportunities to eat with the family and interact with them.^40^ Finally, although the COVID‐19 pandemic has changed our lives and work, it has made people more concerned about their health. Through the pandemic, people are becoming more aware of the health benefits of a good lifestyle and use this opportunity to rebuild a healthy life.

The COVID‐19 pandemic could affect health care systems and adversely affect the screening and management of diabetes. For example, a primary care study in England found a 70% decline in diabetes diagnoses and a 30% decline in HbA1c detection rates during April 2020, which did not return to normal levels until 6 months later.^41^ GDM affects more than 20 million live births worldwide and increases the risk of adverse pregnancy outcomes.^12^ Controlling glycemia in pregnant women with GDM can reduce the occurrence of adverse pregnancy outcomes. For example, lifestyle changes can reduce the incidence of infants who are large for gestational age by approximately 50% in pregnant women with GDM and reduce the risk of obstructed shoulder birth.^42^, ^43^ Our findings show that the COVID‐19 pandemic does not affect pregnancy outcomes in pregnant women with GDM. After the COVID‐19 pandemic, medical personnel has been helping pregnant women to do self‐monitoring and family protection by providing services such as pregnancy health promotion and pregnancy health consultation guidance through online platforms such as WeChat, telephone, Weibo, and online maternity school. The experience from China has shown that during the COVID‐19 pandemic, there was little impact on pregnancy outcomes with the help of telemedicine, despite a decrease In patient visits.^22^ Telemedicine is as effective as conventional care in managing GDM, reducing pregnancy complications, and improving maternal and neonatal glycemia.^44^, ^45^, ^46^ During the COVID‐19 pandemic, the use of telemedicine by medical professionals to instruct women with GDM on self‐care was beneficial and could be effective in managing complications of GDM.^47^ Moreover, pregnant women with GDM are more satisfied with telemedicine than with traditional care, and there is no cost difference between the two options.^45^ These findings suggest telemedicine can be used to manage pregnant women with GDM in the current setting.

Our study was grouped more scientifically than previously published articles.^19^ In addition, we explored changes in GDM prevalence in the prepandemic, pandemic, and postpandemic eras of COVID‐19, which no other study has done. However, we must acknowledge the limitations of this study. First, we included data from only one center. Therefore, due to the varying severity of the COVID‐19 pandemic, our findings may not be generalizable to other regions or countries. Second, we could not obtain the number of outpatient visits but instead used the number of pregnant women who underwent OGTT in the outpatient setting, which may have caused some bias. After the COVID‐19 pandemic, transportation restrictions or local policies caused some pregnant women to complete this test locally. Finally, blood glucose is the diagnostic criterion for GDM, so the accuracy of the test results is essential. Unfortunately, it is not’ easy to guarantee accurate results every time. To ensure the accuracy of test results, our laboratory performs daily internal quality control and regularly participates in external quality assurance programs.

## 5 CONCLUSIONS

Based on current data, we found that after the COVID‐19 pandemic, the prevalence of GDM increased during the worst part of the epidemic, but the overall prevalence of GDM decreased each year. In addition, the pandemic did not change the risk of adverse pregnancy outcomes in pregnant women with GDM. Although the COVID‐19 pandemic in China is under control, the global COVID‐19 pandemic continues. Therefore, we need to continuously monitor and explore the association of the COVID‐19 pandemic with GDM to understand better the pandemic's impact on maternal and infant health.

## AUTHOR CONTRIBUTIONS

Binbin Yin, Kaiqi Wu, Xiuzhi Duan, and Bo Zhu were involved in the design, investigation, data collection and analysis, and manuscript writing. Lingwei Hu, Wanlu Zheng, and Yidan Zheng were involved in data collection and analysis. All authors have agreed on the final version of the manuscript.

## CONFLICT OF INTEREST

The authors declare that there are no conflicts of interest regarding the publication of this paper.

## INFORMED CONSENT

For this type of study, formal consent is not required.

## REGISTRY AND THE REGISTRATION NO. OF THE STUDY/TRIAL

N/A.
